# Promotion of Human Early Embryonic Development and Blastocyst Outgrowth *In Vitro* Using Autocrine/Paracrine Growth Factors

**DOI:** 10.1371/journal.pone.0049328

**Published:** 2012-11-12

**Authors:** Kazuhiro Kawamura, Yuan Chen, Yimin Shu, Yuan Cheng, Jie Qiao, Barry Behr, Renee A. Reijo. Pera, Aaron J. W. Hsueh

**Affiliations:** 1 Program of Reproductive and Stem Cell Biology, Department of Obstetrics and Gynecology, Stanford University School of Medicine, Stanford, California, United States of America; 2 Department of Obstetrics and Gynecology, Akita University Graduate School of Medicine, Akita, Japan; 3 Center of Reproductive Medicine, Department of Obstetrics and Gynecology, Peking University Third Hospital, Beijing, People’s Republic of China; Institute of Zoology, Chinese Academy of Sciences, China

## Abstract

Studies using animal models demonstrated the importance of autocrine/paracrine factors secreted by preimplantation embryos and reproductive tracts for embryonic development and implantation. Although *in vitro* fertilization-embryo transfer (IVF-ET) is an established procedure, there is no evidence that present culture conditions are optimal for human early embryonic development. In this study, key polypeptide ligands known to be important for early embryonic development in animal models were tested for their ability to improve human early embryo development and blastocyst outgrowth *in vitro*. We confirmed the expression of key ligand/receptor pairs in cleavage embryos derived from discarded human tri-pronuclear zygotes and in human endometrium. Combined treatment with key embryonic growth factors (brain-derived neurotrophic factor, colony-stimulating factor, epidermal growth factor, granulocyte macrophage colony-stimulating factor, insulin-like growth factor-1, glial cell-line derived neurotrophic factor, and artemin) in serum-free media promoted >2.5-fold the development of tri-pronuclear zygotes to blastocysts. For normally fertilized embryos, day 3 surplus embryos cultured individually with the key growth factors showed >3-fold increases in the development of 6–8 cell stage embryos to blastocysts and >7-fold increase in the proportion of high quality blastocysts based on Gardner’s criteria. Growth factor treatment also led to a 2-fold promotion of blastocyst outgrowth *in vitro* when day 7 surplus hatching blastocysts were used. When failed-to-be-fertilized oocytes were used to perform somatic cell nuclear transfer (SCNT) using fibroblasts as donor karyoplasts, inclusion of growth factors increased the progression of reconstructed SCNT embryos to >4-cell stage embryos. Growth factor supplementation of serum-free cultures could promote optimal early embryonic development and implantation in IVF-ET and SCNT procedures. This approach is valuable for infertility treatment and future derivation of patient-specific embryonic stem cells.

## Introduction

Early embryonic development from fertilization to implantation takes place in the oviduct and uterus without direct cell-to-cell contact with reproductive tract tissues until the final stage. During transit through oviduct and uterus, cells in preimplantation embryos undergo division, differentiation, and apoptosis. Early studies using animal models demonstrated enhanced embryonic development and survival when the volume of culture media was reduced [1], [2] or when early embryos were cultured in groups [3], [4] to increase concentrations of locally secreted factors. In addition, promotion of blastocyst formation and inhibition of apoptosis were found when culture media for animal embryos were supplemented with individual growth factors, including insulin-like growth factor-I (IGF-I), epidermal growth factor (EGF), fibroblast growth factor (FGF), platelet derived growth factor (PDGF), brain-derived growth factors (BDNF), artemin, colony stimulating factor 1(CSF1), glial cell-line derived neurotrophic factor (GDNF), and others [1], [2], [3], [4], [5], [6], [7], [8], [9]In addition, the development of in vitro cultured embryos is retarded compared with their counterparts at comparable stages of development in vivo [10] and putative paracrine factors secreted by the reproductive tract were shown to enhance early embryonic development [11].

Recent progress in sequential culture media allowed extended culture of human embryos to the blastocyst-stage and blastocyst transfer is effective in selecting high-quality embryos for successful pregnancy [12], leading to the birth of several million IVF babies [14], [15]. However, the efficiency of human blastocyst development *in vitro* remains to be improved. Several studies using surplus human material suggested the promotion of blastocyst development *in vitro* when culture media were supplemented with growth factors, including EGF [13], IGF-I [14], BDNF [18], and granulocyte macrophage colony-stimulating factor (GM-CSF) [19], [20]. Because most routine human embryo cultures do not contain growth factors, we hypothesized that inclusion of autocrine/paracrine growth factors in the culture media could improve early embryonic development. We investigated the expression of key ligand-receptor pairs in cleavage-stage human embryos derived from tri-pronuclear zygotes and specific ligands in human uterine endometrium. We individually cultured abnormally fertilized zygotes and normally fertilized day 3 embryos from IVF programs in micro-drops using routine serum-free culture media supplemented with a group of key growth factors to improve embryo development. We examined if these growth factors could improve blastocyst outgrowth *in vitro* using normally fertilized hatching blastocysts. Because the development of reconstructed embryos after SCNT is sub-optimal, we also tested if supplementation of culture media with key growth factors could increase the development of SCNT-derived embryos.

## Materials and Methods

### Source of Donated Embryos, Oocytes, and Endometrium

We have used three sources of human oocytes/embryos to evaluate the effects of autocrine/paracrine factors on early embryonic development *in vitro*, including abnormally fertilized zygotes, normally fertilized day 3 and 5 embryos, and reconstructed embryos after SCNT. We also obtained human endometrium for expression analyses. Informed signed consent from patients and approval from the Human Subject Committees of Stanford University School of Medicine, The Third Hospital, Peking University, and the Akita University Graduate School of Medicine were obtained.

A total of 88 abnormally fertilized tri-pronuclear zygotes from 56 patients (32.1±2.6 years of age) undergoing IVF-ET at the Third Hospital, Peking University were obtained. These abnormal zygotes were allowed to develop to the cleavage stage (6–10-cell-stage) before fixation for immunofluorescence staining of ligand-receptor pairs or directly used for *in vitro* cultures with growth factors. For normally fertilized human early embryos, a total of 153 and 81 cryo-preserved surplus human day 3 and 5 embryos donated by 25 (35.4±4.6 years of age) and 55 patients (38.9±5.2 years) to the RENEW Biobank at Stanford University School of Medicine and Akita University Graduate School of Medicine, respectively, were thawed for *in vitro* cultures. In addition, a total of 63 failed-to-be-fertilized oocytes donated for SCNT from patients at the IVF program of Stanford University were vitrified by using hemi-straw as a carrier and stored in liquid nitrogen for SCNT experiments. All abnormal and surplus normal embryos were obtained from patients following informed consent and institutional approval.

### Immunofluorescence Staining of Growth Factors and Their Receptors in Human Embryos

Cleavage-stage embryos derived from tri-pronuclear zygotes were fixed with 4% paraformaldehyde for 30 min at 23°C. After permeabilization with 0.1% Triton X-100, embryos were pre-incubated in 5% BSA for 1 h before incubation with specific primary antibodies diluted in PBS supplemented with 1% BSA overnight at 4°C. The antibodies used include: anti-EGF (Abcam, Cambridge, MA), anti-IGF-I (Santa Cruz Biotech, Santa Cruz, CA), anti-GM-CSF (Abcam), anti-BDNF (Santa Cruz Biotech), anti-CSF-1 (Abcam), anti-artemin (Abcam), anti-GDNF (Abcam), anti-EGF receptor (Abcam), anti-IGF-I receptor (Abcam), anti-GM-CSF receptor (Santa Cruz Biotech), anti-TrkB (R&D systems, Minneapolis, MN), anti-CSF-1 receptor (Santa Cruz Biotech), Anti-GFR alpha 3 antibody (Abcam), After three washes in PBS containing 0.1% Tween 20, embryos were incubated with fluorescein isothiocyanate-conjugated secondary antibodies (Jackson Labs, West Grove, PA) for 1 h at 23°C. Nuclear status of embryos was evaluated by staining with 10 µg/ml propidium iodide for 10 min and examined with a confocal laser-scanning microscope (Zeiss LSM 510, Jena, Germany). For negative controls, the primary antibody was replaced with non-immune IgG.

### RT-PCR Analyses and Immunostaining of Growth Factors Expression in the Human Endometrium

Human endometrial samples at secretory phase were obtained from five patients, aged 36–42 years, who underwent gynecological surgery for uterine leiomyoma and exhibited normal menstrual cycles. The menstrual stage was confirmed based on urinary LH levels and ovarian ultrasonography. The expression of key growth factors in secretory phase endometrium was determined by conventional RT-PCR and immunostaining. For conventional RT-PCR, specific primers for growth factors and β-actin were used (Table S1). PCR reactions comprised 30 cycles of amplification with denaturation at 94°C for 30 sec, annealing at 60°C for 30 sec, and elongation at 72°C for 30 sec. For negative controls, no mRNA was included. To confirm identity, the PCR products were sequenced as described [21].

For immunostaining, human endometrial samples at secretory phase were fixed with 10% neutral buffered formalin overnight at 23°C. Tissues were embedded in paraffin and sectioned at 5-µm intervals. After deparaffinization and dehydration, antigen retrieval was performed by autoclave heating at 121°C in 10 mM citrate buffer for 10 min. Endogenous peroxidase activities were quenched with 0.3% hydrogen peroxidase. After blocking with 10% BSA-Tris-buffered saline (TBS) for 30 min, slides were incubated with specific antibodies overnight at 4°C. After three washes, slides were incubated with biotinylated secondary antibodies (Invitrogen, Carlsbad, CA) for 30 min at 23°C. After three washes, bound antibodies were visualized using a Histostain SP kit (Invitrogen). For negative controls, the primary antibody was replaced by nonimmune IgG.

### *In vitro* Embryo Cultures

Tri-pronuclear zygotes were cultured individually in 30 µl microdrops containing the Global-Medium (G-M, LifeGlobal, Guilford, CT) with 5% human serum albumin in the presence or absence of EGF, IGF-I, GM-CSF, BDNF, and CSF-1 (PeproTech, Rocky Hill, NJ), all at 10 ng/ml. The culture medium was renewed every 48 h. Embryonic development was evaluated at 96, 120, and 144 h after culture.

Normally fertilized embryos frozen on day 3 of culture by slow cooling were thawed by using a 2-step thawing protocol [15]. Poor-quality embryos (un-cleaved, retarded growth, and severely fragmented) were discarded and good-quality embryos were selected according to the Veeck’s criteria [16] and subdivided into two groups: optimal group (>6-cell-stage, grade 1 or 2), and suboptimal group (>6-cell-stage, grade 3; 3- to 5-cell-stage, grade 1 to 3). Embryos were then transferred to the MultiBlast Medium (Irvine Scientific, Santa Ana, CA) with 10% Serum Substitute Supplement (SSS, Irvine Scientific, Santa Ana, CA) and further cultured at 37°C with 5% CO_2_, 5% O_2_ and 90% N_2_ with or without growth factor mixtures containing 10 ng/ml of EGF, IGF-I, GM-CSF, BDNF, CSF-1, artemin, and GDNF (R&D Systems). Individual embryos were cultured for 72 h in a 30 µl drop of medium and their development was evaluated. The doses of these growth factors chosen for these experiments were based on previous studies (BDNF [21], GDNF [24], artemin [8], EGF [13], IGF-I [14], GM-CSF [17], CSF-1 [9]).

### Real-time Quantitative RT-PCR (RT-qPCR) Analyses of Growth Factors/receptors Expression in Blastocysts and Blastocyst Adhesion and Outgrowth Assays

Normally fertilized embryos were frozen on day 5 of culture by vitrification using a Cryotop vitrification kit (KITAZATO BioPharma, Shizuoka, Japan) [18]. Among surplus frozen embryos, high quality blastocysts (3AA to 5 AA) based on Gardner’s criteria were thawed by using a Cryotop thawing kit (KITAZATO BioPharma) [18] and used for real-time RT-qPCR to determine the expression of growth factors and their receptors. Some blastocysts were subjected to blastocyst adhesion and outgrowth assays to evaluate the effects of the growth factors on implantation.

Real-time RT-qPCR of transcript levels in blastocysts was performed using a SmartCycler (Takara, Tokyo, Japan) [7], [19], [20] with primers listed in Table S2. To determine the absolute copy number of target transcripts, cloned plasmid cDNAs for individual gene were used to generate a calibration curve. Purified plasmid cDNA templates were measured, and copy numbers were calculated based on absorbance at 260 nm. A calibration curve was created by plotting the threshold cycle against the known copy number for each plasmid template diluted in log steps from 10^5^ to 10^1^ copies. Each run included standards of diluted plasmids to generate a calibration curve, a negative control without a template, and samples with unknown mRNA concentrations. Data were normalized based on β-actin transcript levels.

Blastocyst adhesion and outgrowth was assayed using a procedure established by Armant et al. [21]. Thawed embryos were then cultured individually in 30 µl microdrops of in the BlastAssist medium (MediCult, Måløv, Denmark) for 48 h until they started to hatch. Individual hatching embryos were transferred to a single well of a 24-well plate coated with 200 µl of growth factor-reduced Matrigel (Becton Dickinson Labware, Oxford, UK) overlaid with 400 µl of the BlastAssist medium with or without growth factor mixtures containing 10 ng/ml of EGF, IGF-I, GM-CSF, BDNF, CSF-1, artemin, and GDNF. Blastocysts that adhered to the culture plate were designated as adhesion blastocysts. Immunostaining with cell markers indicated that the cells undergoing outgrowth were trophoblasts since they showed immunoreactivity for cytokeratin but were negative for vimentin and Dolichos biflorus agglutinin (markers for ICM-derived cells). When trophoblast cells had grown outward from the adhered blastocysts and the primary trophoblast cells became visible, these embryos were designated as outgrowth blastocysts. The proportions of blastocysts undergoing adhesion and outgrowth were estimated at 72 h after growth factor treatment [27], [28]. The proportions of hatched blastocysts showing adhesion or outgrowth were used to estimate the implantation capacity of blastocysts *in vitro*.

### SCNT and Subsequent Embryo Cultures

Vitrification of failed-to-be-fertilized oocytes was performed using hemi-straws with a vitrification kit (CooperSurgical Inc., Trumbull, CT) as described [22]. Human skin fibroblasts were cultured in 0.5% serum for 48 h to arrest the cell cycle at G_0_/G_1_ stage. To improve the accuracy of enucleation, removal of meiotic spindle was performed under an OoSight imaging system (CRI, Woburn, MA). After thawing cryopreserved oocytes using a warming kit (CooperSurgical Inc.), a single fibroblast was injected into the cytoplasm of each enucleated oocyte. After 2 h of culture, the reconstructed oocytes were chemically activated using 5 µM calcium ionophone (Sigma, St. Louis, MO) for 10 min, followed by incubation for 4 h with 2.5 mM 6-dimethylaminopurine (Sigma), a serine/threonine protein kinase inhibitor. Activated reconstructed oocytes were then cultured individually in 30 µl microdrops of SAGE blastocyst medium with 10% SSS the presence or absence of growth factor mixtures containing 10 ng/ml of EGF, IGF-I, GM-CSF, BDNF, CSF-1, artemin, and GDNF. The culture medium was renewed every 48 h with embryonic development evaluated daily for up to 144 h.

### Statistical Analysis

Statistical analysis was performed using the chi-square test with *P*<0.05 representing significant differences. Comparisons of data were performed in the same Center for embryos cultured using the same type of culture medium with or without supplementation of growth factors.

## Results

### Expression of Ligand-receptor Pairs of Key Growth Factors in Human Triploid Embryos and Endometrium

**Figure 1:**
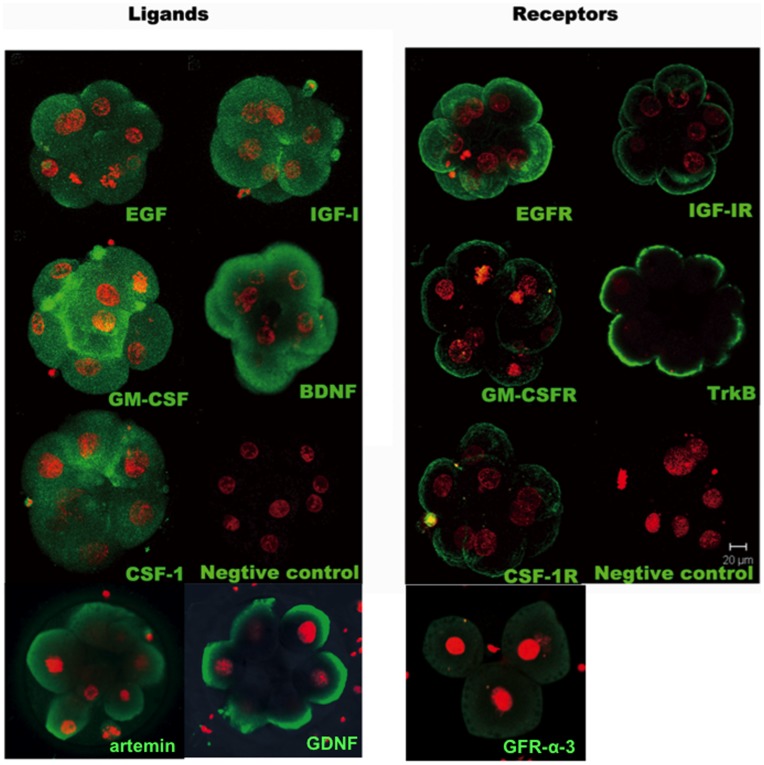
Immunofluorescence staining of polypeptide ligand-receptor pairs of key growth factors in triploid human embryos. Tri-pronuclear zygotes discarded from the IVF program were cultured to generate cleavage stage embryos (3–10 cell-stage) for immunostaining using specific antibodies against different ligands and receptors. Green signals for ligand/receptor pairs (EGF/EGF receptor, IGF-1/IGF-1 receptor, GM-CSF/GM-CSF receptor, BDNF/TrkB, CSF-1/CSF-1 receptor, atermin, GDNF/Anti-GFR alpha 3) were found following staining with specific antibodies. Embryonic nuclei were stained with propidium iodide (red signals). Negative controls were incubated with nonimmune IgG. Bar = 20 µm.

We collected tri-pronuclear zygotes discarded from the IVF program and cultured them to the cleavage-stage embryos for analyses. As shown in Fig. 1, immunostaining using specific antibodies against key ligand-receptor pairs demonstrated the expression of different growth factors (EGF, IGF-I, GM-CSF, BDNF, CSF-1, artemin, and GDNF) in the cytoplasm of tri-pronuclear embryos. Receptors for these ligands (EGF receptor, IGF-I receptor, GM-CSF receptor, TrkB, CSF-1 receptor, and GFR alpha 3) were also expressed in these embryos with signals found in the plasma membrane.

**Figure 2:**
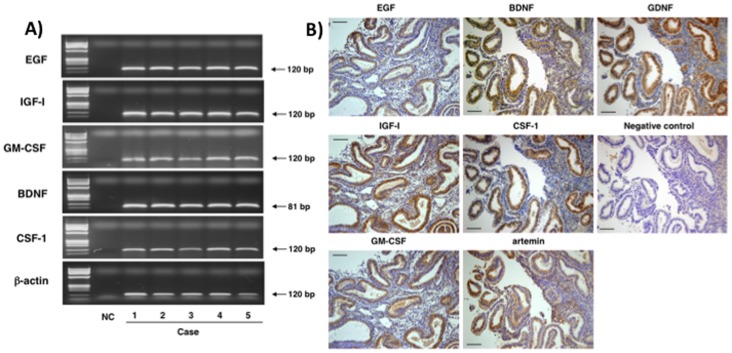
Expression of key growth factors in human endometrium. Human endometrial samples were obtained from five different patients at the secretory phase of their cycle. A) Gel electrophoretic analyses of RT-PCR products for different growth factors. Arrows indicate the expected sizes of the amplified PCR products. bp: base pair, B) Immunostaining analyses of growth factor expression in human endometrium. Brown signals for growth factors (EGF, IGF-1, GM-CSF, BDNF, CSF-1, artemin, and GDNF) were found following staining with specific antibodies. Negative controls were incubated with nonimmune IgG. Bar = 100 µm.

We further analyzed the expression of key growth factors in human endometrium obtained from patients at the secretory phase of the cycle by RT-PCR. As shown in Fig. 2A, gel electrophoresis indicated the amplification of PCR products of the predicted sizes for different growth factors, including EGF, IGF-I, GM-CSF, BDNF, and CSF-1. In addition, immunostaining analyses confirmed strong protein expression of these growth factors as well as artemin and GDNF in glandular epithelium of uterine endometrium with weak signals found in stromal cells (Fig. 2B).

### Supplementation of Culture Media with Key Autocrine/paracrine Growth Factors Promoted the Development of Tri-pronuclear Zygotes to Blastocysts

**Figure 3:**
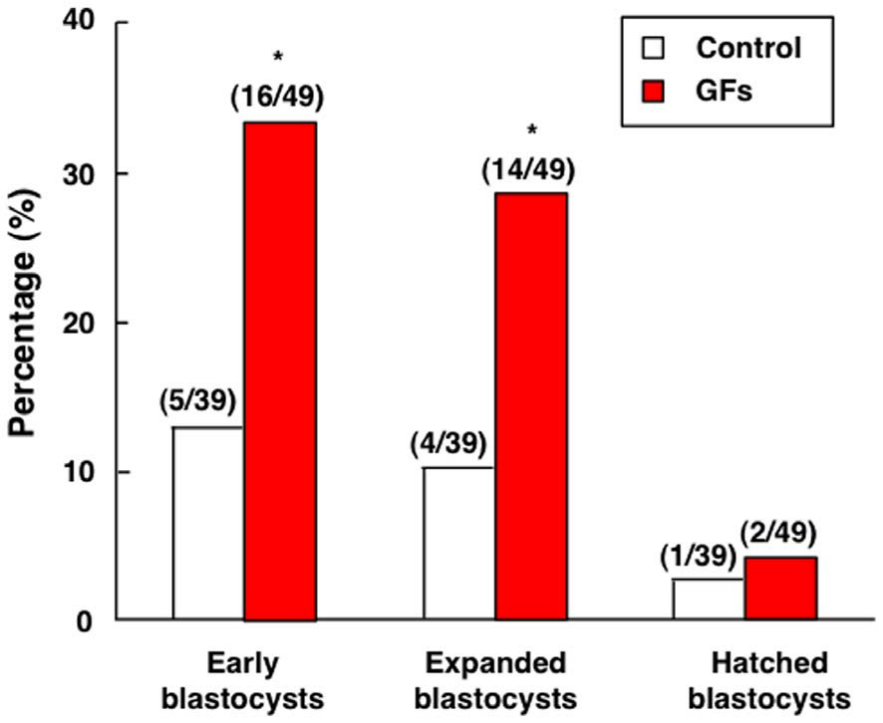
Effects of growth factor treatment on the development of cultured tri-pronuclear zygotes. Human tri-pronuclear zygotes were cultured individually in micro-drops containing serum-free media with or without key growth factors for up to 144 h. Morphological changes were monitored for blastocyst formation at early, expanded, and hatched stages. Numbers of embryos developed to specific stages vs. total number of embryos studied are listed on top of each column. *, *P*<0.05 vs. control. GFs: growth factors.

We cultured individual tri-pronuclear zygotes with or without different growth factors (EGF, IGF-I, GM-CSF, BDNF, and CSF-1) for up to 144 h before evaluation of their developmental potential. As shown in Fig. 3, the addition of growth factors to the culture media increased the development of tri-pronuclear zygotes to early and expanded blastocyst stages by 2.5- and 2.8-fold, respectively. However, few of these abnormal embryos developed to the hatched blastocyst stage with or without growth factor supplementation.

### Supplementation of Culture Media with Key Growth Factors Promoted Blastocyst Formation of Cryopreserved Day 3 Embryos and Increased the Proportion of High Quality Blastocysts

**Figure 4:**
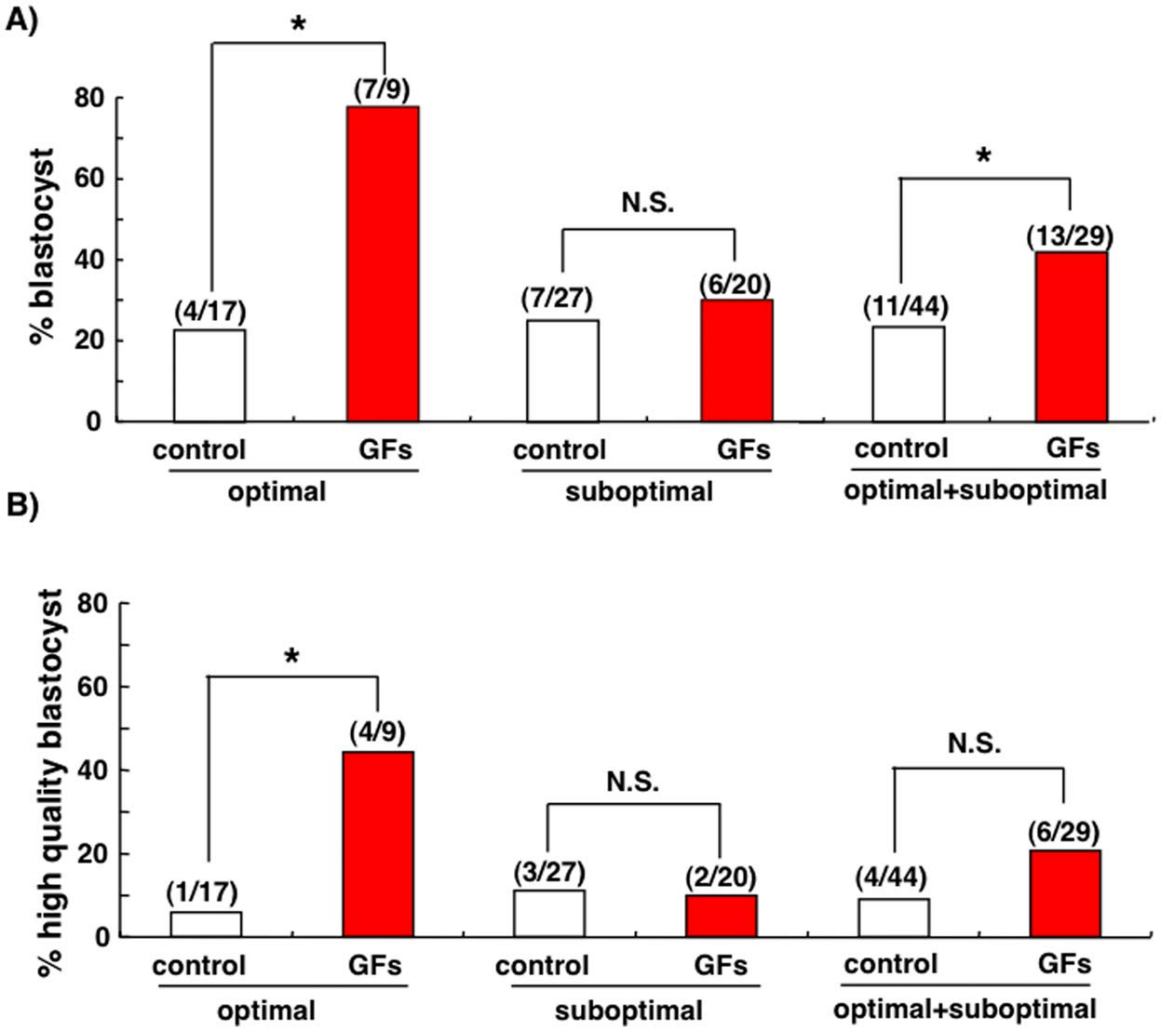
Treatment with autocrine/paracrine growth factors promoted the development of day 3 human embryos to the blastocyst stage. Cryopreserved human day 3 embryos were thawed and evaluated based on their morphology. After discarding poor-quality embryos, good-quality embryos were divided into optimal (>6-cell-stage, grade 1 or 2) and suboptimal groups (>6-cell-stage, grade 3; 3- to 5-cell-stage, grade 1 to 3). Embryos were then cultured with or without key growth factors for 72 h in micro-drops of medium. At the end of culture, the proportion of blastocyst formation was evaluated. Numbers inside parentheses indicate blastocysts/total embryos for each group. *, *P*<0.05; N.S., no significant differences. A) Development of all blastocysts. B) Development of high quality blastocysts scored as 3AA to 5 AA.

Cryopreserved day 3 embryos were thawed and evaluated based on their morphology. After discarding fragmented poor-quality embryos, good-quality embryos were divided into optimal (>6-cell-stage, grade 1 or 2) and suboptimal groups (>6-cell-stage, grade 3; 3- to 5-cell-stage, grade 1 to 3) based on the Veeck’s criteria [16]. These embryos were allocated at random and then cultured with or without different growth factors (EGF, IGF-I, GM-CSF, BDNF, CSF-1, artemin, and GDNF) for 72 h before evaluation of their developmental potential. As shown in Fig. 4A, a 3.3-fold increase in the proportion of blastocyst-stage-embryos was found after treatment of embryos of the optimal group with growth factors. In contrast, treatment of the suboptimal embryos with growth factors did not affect blastocyst formation (P>0.05). We further evaluated the formation of high quality blastocysts (3AA to 5 AA) based on Gardner’s criteria [23]. As shown in Fig. 4B, a 7.6-fold increase in high quality blastocysts was found between control and growth factor-treated groups. Again, treatment with growth factors did not increase high quality blastocysts derived from suboptimal embryos and a 2-fold increase in high quality blastocyst formation was found when all embryos were evaluated.

### Supplementation of Culture Media with Key Growth Factors Promoted Blastocyst Outgrowth

**Figure 5:**
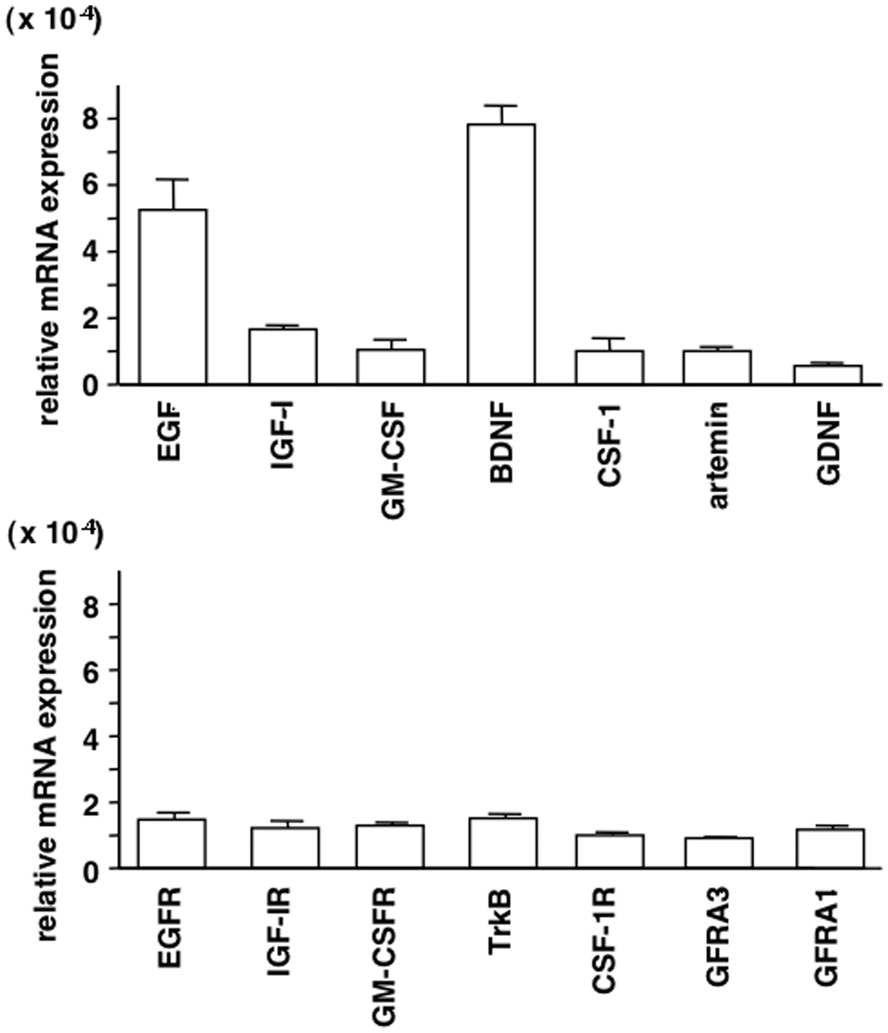
Expresssion of key growth factors and receptors in human blastocysts. Cryopreserved human day 5 embryos were used for quantitative RT-PCR analyses of transcript levels for different ligand-receptor pairs (EGF/EGFR, IGF-I/IGF-IR, GM-CSF/GM-CSFR, BDNF/TrkB, CSF-1/CSF-1R, artemin/GFRA3, and GDNF/GFRA1). Levels of all mRNAs were normalized based on those for β-actin in the same sample (mean ± SEM, n = 3).

**Figure 6:**
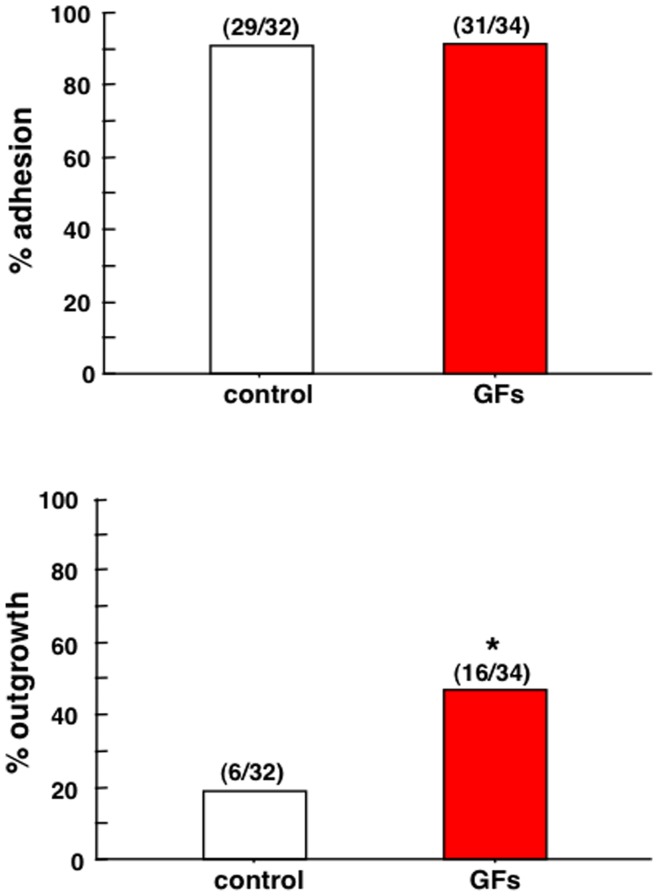
Treatment with autocrine/paracrine growth factors promoted blastocyst outgrowth. Cryopreserved human day 5 embryos were thawed and cultured for 48 h until hatching. High quality hatching embryos were then cultured with or without growth factors for 72 h in a well coated with Matrigel. At the end of culture, the proportion of blastocyst adhesion and outgrowth was evaluated. Numbers inside parentheses indicate blastocyst adhesion or outgrowth/total embryos for each group. *, *P*<0.05.

To evaluate the roles of ligand-receptor pairs of key growth factors in blastocyst implantation, their expressions in thawed cryopreserved day 5 embryos were determined by real-time RT-qPCR. As shown in Fig. 5, all ligand-receptor pairs were expressed in blastocysts. Among the ligands, the levels of EGF and BDNF were high (Fig. 5, upper panel), suggesting possible dominant autocrine actions, whereas transcript levels of the receptors were comparable (Fig. 5, lower panel). Thawed cryopreserved day 5 embryos were cultured for 48 h to obtain hatching embryos before analyses of blastocyst adhesion and outgrowth *in vitro*. Embryos were cultured in a well coated with Matrigel with or without different growth factors (EGF, IGF-I, GM-CSF, BDNF, CSF-1, artemin, and GDNF) for 72 h before evaluation of their implantation potential. As shown in Fig. 6, the addition of growth factors to the culture media increased blastocyst outgrowth by 2.5-fold. In contrast, treatment with growth factors did not affect blastocyst adhesion.

### Supplementation of Culture Media with Key Growth Factors Promoted the Development of SCNT Embryos

After warming, vitrified failed-to-be-fertilized oocytes served as recipients for SCNT using serum-starved (48 h) fibroblasts as the donor karyoplast. To confirm that the pronuclear formed after artificial activation was from injected fibroblast, we observed mitotic spindle 2 h following fibroblast injection with the assistance of OoSight. As shown in Fig. S1, only one birefringent spindle was seen in the center of each reconstructed oocyte. Among the 18 activated oocytes, 17 showed only one pronuclear in the cytoplasm. Because the development of reconstructed embryos after SCNT is suboptimal, we tested the potential effects of supplementing culture media with different growth factors (EGF, IGF-I, GM-CSF, BDNF, CSF-1, artemin, and GDNF). As shown in Table 1, ∼90% of oocytes survived after vitrification-warming, among which ∼50% of them survived subsequent enucleation and fibroblast injection procedures. Following successful nuclear transfer, early embryonic development was improved with growth factor supplementation of culture media as reflected by the development of >5-fold increases in the proportion (40%, 6/15) of cultured SCNT oocytes to embryos with more than 4-cell stage embryos as compared those without growth factor supplementation (7%, 1/14). Although with low efficiency, the most advanced SCNT embryo reached the 16-cell stage when cultured in media supplemented with growth factors.

**Table 1:** Development of SCNT embryos cultured in media with or without growth factor supplementation.

	Without growth factors	With growth factors
Vitrified oocytes	34	29
Oocytes surviving the thawing	31	25
Oocytes used for enucleation	29	24
No. of successful enucleation	21	22
No. of successful fibroblast injection	14	15
No. of activated oocytesNo. of cleaved embryos	65	128
Embryos with ≥4-cells on day 3	1	6*
Embryos with >8-cells	0	1(16-cell)

Failed-to-be-fertilized oocytes were vitrified and then thawed before evaluation for morphology and SCNT using serum-starved fibroblasts arrested at the G_0_/G_1_ stage. After SCNT, reconstituted oocytes were activated using using 5 µM calcium ionophone for 10 min,followed by incubation for 4 h with 2.5 mM 6-dimethylaminopurine, and cultured in media with or without growth factors. Numbers of oocytes/embryos at each experimental stage are shown. *P = 0.187.

## Discussion

Following culturing individual human embryos in chemically defined serum-free media, we demonstrated the ability of key autocrine/paracrine growth factors to promote early embryonic development and implantation. Treatment with key growth factors enhanced the development of abnormal tri-pronuclear zygotes, normally fertilized human embryos, and reconstructed embryos following SCNT. The key growth factors not only stimulated embryo growth but also increased the proportion of morphologically good blastocysts, suggesting improvement of embryo quality. Although the facilitatory effects of individual growth factors on human embryo development have been reported [16], [17], [19], [20], [31], [32], the present use of multiple growth factors likely exerts overlapping and redundant actions to allow optimal early embryo development. Culturing of individual embryos further minimizes cross-interaction of embryos and provides the basis to monitor the functions of single high quality embryos for transfer. Demonstration of the facilitatory effects of key growth factors to promote blastocyst outgrowth further provides future opportunity to include them in embryo transfer media to improve implantation success.

Our immunofluorescence staining and real-time RT-qPCR analyses confirmed the expression of key ligand-receptor pairs in human early embryos, underscoring their importance as autocrine/paracrine factors. For each ligand-receptor pairs, ligand antigens were found in the cytoplasm of blastomeres whereas the receptor antigens were found in the plasma membrane, suggesting the secretion of these paracrine/autocrine ligands to act on membrane receptors in an autocrine/paracrine manner. As a 3-day-old human embryo enters the uterus at the morula stage, further development could also be regulated by paracrine factors secreted by the endometrium. Our RT-PCR and immunostaining studies confirmed the expression of these key growth factors in the human endometrium, suggesting their paracrine roles in support of embryo growth after the morula stage.

Earlier studies indicated that tri-pronuclear zygotes are capable of developing into blastocysts albeit with lower efficiency [24]. Taking advantage of the availability of discarded human tri-pronuclear zygotes in IVF, we developed them into cleavage-stage embryos for analyzing the expression of different ligand-receptor pairs known to play autocrine/paracrine functions in animal embryos. We also demonstrated that culturing these abnormally fertilized embryos in serum-free culture media supplemented with growth factors substantially promoted their development by more than 2-fold.

The improvement of sequential culture systems for human IVF during the last decades has allowed extended culture of human early embryos to the blastocyst stage. Blastocyst transfer facilitates the selection of the best embryos with high implantation potential and therefore reduces the number of transferred embryos to avoid multiple pregnancies. However, the current human embryo culture system is still suboptimal and many embryos cannot develop to the blastocyst stage. Our results using normally fertilized day 3 embryos suggest that key autocrine/paracrine growth factors are beneficial to human embryonic development *in vitro*. These growth factors not only increase the rate of blastocyst formation, but also the quality of blastocysts. Indeed, culturing good-quality day 3 embryos in culture medium supplemented with these growth factors resulted in a 3.3-fold increase in the blastocyst formation rate and a 7.6-fold increase in the proportion of high quality blastocysts as compared to controls. These findings are consistent with the hypothesis that autocrine/paracrine factors secreted by early embryos are diluted during culture and growth factor supplementation is necessary to promote optimal blastocyst formation. Selective single blastocyst transfer in patients with good prognosis has been shown to be effective in reducing multiple pregnancies without compromising the pregnancy rate [25]. Because most of the commercially available, chemically-defined media for human embryo cultures in IVF-ET do not contain growth factors, the present supplementation of widely used culture media with autocrine/paracrine growth factors has practical value in future IVF-ET procedures.

Different from previously published reports showing small stimulatory effects of individual growth factors on human embryo development, our combined treatment with several autocrine/paracrine factors showed a robust stimulation of normally fertilized day 3 embryos likely due to additive effects of different growth factors in the promotion of early embryonic development. Inclusion of IGF-I [14] or GM-CSF [17] increased the proportion of embryos developing to the blastocyst stage by 1.51-fold and 2.53-fold, respectively. In our study, treatment embryos with the growth factor cocktail showed a 3.3-fold increase in the proportion of blastocyst-stage-embryos. The ability of these paracrine/autocrine factors to promote development of early human embryos is consistent with findings showing zygote genome activation in human embryos at 4- to 8-cell stages on day 3 after fertilization when the expression of these growth factors begun to increase [26]. In the present combination treatment protocol, several distinct signaling pathways could be activated by the autocrine/paracrine factors used: EGF, IGF-I and BDNF bind to respective receptor tyrosine kinases to activate downstream phophotidyinositol-3-kinase-Akt signaling, CSF1 and GM-CSF interact with type I cytokine receptors to activate the downstream JAK/STAT pathway, whereas GDNF and artemin interact with glycosylphosphatidyl- inositol-anchored receptors to activate downstream cRET and Src kinase pathways [27]. Although the fresh tri-pronuclear zygotes used here were treated with five growth factors due to reagent availability, thawed normally-fertilized and SCNT embryos were treated with seven growth factors. It is likely that these divergent pathways exert overlapping and redundant actions on early embryo development and not all growth factors are needed for optimal embryo growth.

Successful implantation of the blastocyst is essential for reproduction. Implantation of blastocysts is a well-organized process regulated by multiple growth factors and cytokines [28]. We demonstrated the facilitatory effects of key growth factors to promote blastocyst outgrowth. The trophectoderm cells of blastocysts differentiate during embryonic development to form the invasive trophoblasts that mediate implantation of embryos into the uterine wall. The outgrowth of trophoblast cells from cultured blastocysts is believed to reflect the proper differentiation of the embryo, important for trophoblast invasion of the endometrial stroma during implantation in utero [38,39]. Although blastocyst transfer is effective to select the best quality embryos with high implantation potential, overall implantation rate is ∼30% [29], suggesting human embryo transfer might be improved. Due to the low amount of liquid in the uterine cavity, factors included in the transfer media could be retained in high concentrations. Indeed, embryo transfer in medium containing hyaluronan is effective in improving implantation rates in patients with recurrent implantation failure [30], [31], [32].Hyaluronan is the major glycosaminoglycan present in follicular, oviductal and uterine fluids and presumably promotes embryo–endometrial interactions during the initial phases of implantation. Because key growth factors promoted blastocyst outgrowth *in vitro*, future supplementation of embryo transfer media with key growth factors could also promote implantation during embryo transfer.

Generating an autologous patient-specific embryonic stem cell line from SCNT embryos holds great promise for the treatment of degenerative human diseases. Successful derivation of embryonic stem cell lines following SCNT has been reported in mouse [44], rabbit [45], and non-human primates [46]. However, the efficiency for the production of embryonic stem cell lines following SCNT is still low (<2%), particularly when adult somatic cells were used as the donor karyoplasts. Although many embryonic stem cell lines have been derived from surplus human blastocysts [47,48], no human cell-lines have been generated following SCNT. Among the many compounding factors, suboptimal *in vitro* culture condition contributes to the poor embryonic development of reconstructed embryos following SCNT. The present study represents an attempt to optimize the culture conditions for the development of human SCNT embryos. Although no blastocyst was obtained following fibroblast nuclear transfer, there was a trend to an augmented development of reconstructed embryos cultured with media containing autocrine/paracrine growth factors. Results from the present study provide the basis for future use of autocrine/paracrine factors to facilitate the derivation of patient-specific embryonic stem cells.

In conclusion, the present study demonstrated the utility of growth factor supplementation for optimal human early embryo development and blastocyst outgrowth. The findings may allow the design of better conditions for individual human embryo cultures, for estimating their developmental potentials using secretory products, and for the inclusion of growth factors in embryo transfer media to promote implantation. Although the present experimental design is based on the supplementation of endogenous growth factors diluted during assisted reproductive procedures, future studies on the potential side effects of these paracrine/autocrine factors on chromosomal numbers, genomic integrity, proteomic changes, and epigenetic modifications are essential before clinical use.

## Supporting Information

Figure S1 **Morphology of reconstructed oocytes.**
(TIF)

Table S1 **Conventional RT-PCR primers for growth factors.**
(TIF)

Table S2 **Quantitative RT-PCR primers for growth factors and receptors.**
(TIF)
