# Supplementary figures and images for: Promotion of Human Early Embryonic Development and Blastocyst Outgrowth In Vitro Using Autocrine/Paracrine Growth Factors
Source: PLoS One. 2012 Nov 12;7(11):e49328. doi: 10.1371/journal.pone.0049328 (PMC3495911; doi:10.1371/journal.pone.0049328)

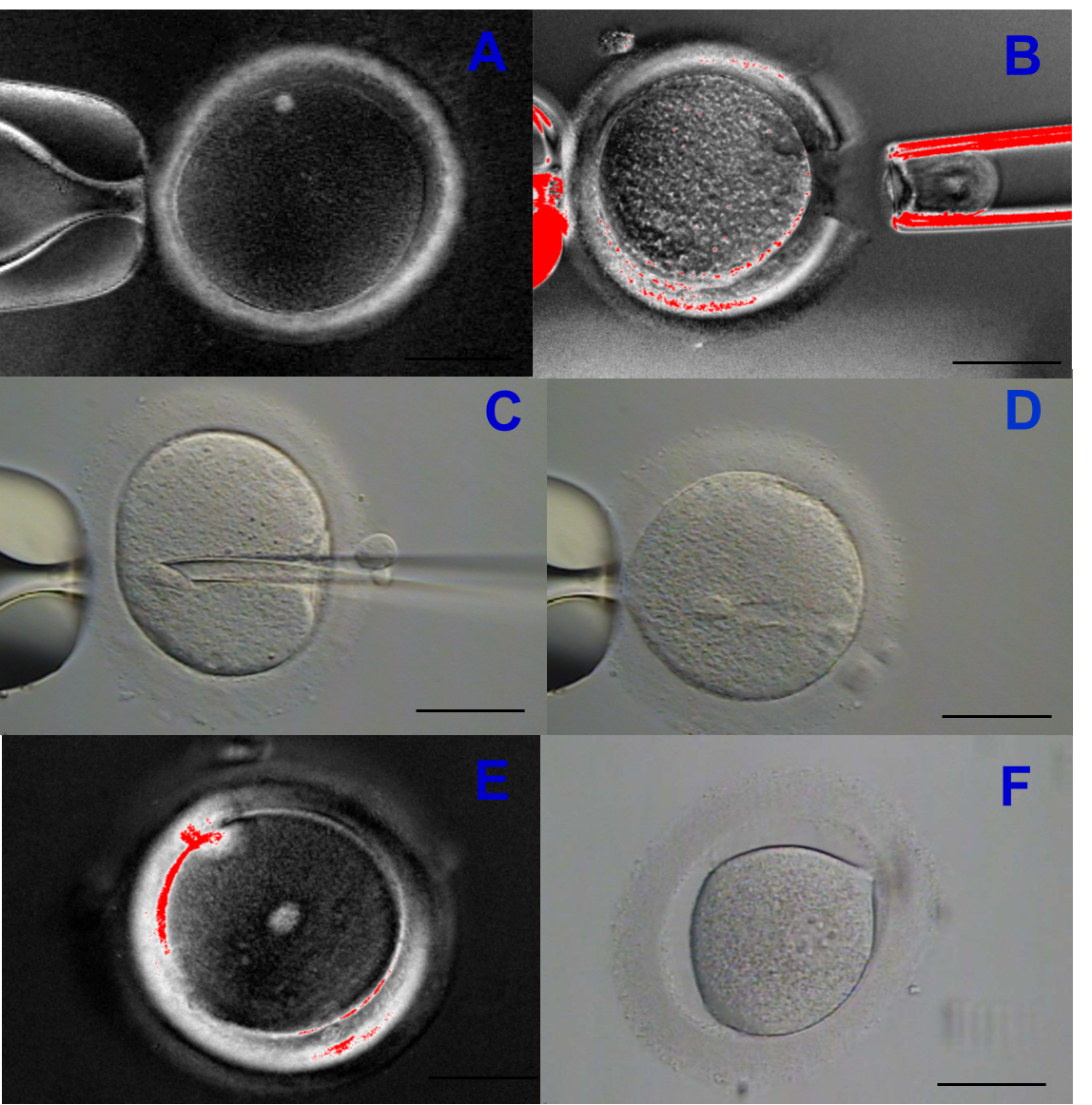

Supplement: Figure S1 — Morphology of reconstructed oocytes. (TIF) [file pone.0049328.s001.tif]
